# Phytochemical Profiling (Phenolics and Carotenoids) and Antioxidant Potential of Apple, Plum, Carrot, and Beetroot Juice Pomaces: Towards Sustainable Food Ingredients

**DOI:** 10.3390/foods15183244

**Published:** 2026-09-14

**Authors:** Maria Simona Chiș, Anca Corina Fărcaș, Gheorghe Adrian Martău, Dan Cristian Vodnar, Anamaria Pop, Adriana Păucean, Simona Maria Man, Paul Andor

**Affiliations:** 1 Department of Food Engineering, University of Agricultural Sciences and Veterinary Medicine Cluj-Napoca, 400372 Cluj-Napoca, Romania; simona.chis@usamvcluj.ro (M.S.C.); anamaria.pop@usamvcluj.ro (A.P.); adriana.paucean@usamvcluj.ro (A.P.); simona.man@usamvcluj.ro (S.M.M.); 2 Department of Food Science, University of Agricultural Sciences and Veterinary Medicine Cluj-Napoca, 400372 Cluj-Napoca, Romania; anca.farcas@usamvcluj.ro (A.C.F.); adrian.martau@usamvcluj.ro (G.A.M.); dan.vodnar@usamvcluj.ro (D.C.V.)

**Keywords:** juice processing by-products, upcycled food ingredients, bioactive compounds, antioxidant activity, circular bioeconomy, pomace valorization, polyphenols

## Abstract

Apple (*Malus domestica* Borkh. cv. Jonathan), beetroot (*Beta vulgaris* L.), carrot (*Daucus carota* L.), and plum (*Prunus domestica* L.) pomaces are among the major by-products generated by the juice processing industry in Europe, particularly in producing countries such as Poland, Germany, Spain, and Romania. Conventionally discarded or utilized as animal feed or bioenergy pellet production, these matrices hold potential as high-value functional food ingredients. Supporting circular bioeconomy goals, this study evaluated the potential of industrial pomaces generated during continuous juice pressing as high-value functional food ingredients following their conversion into powders by air-drying and milling. The pomace powders were characterized for proximate composition, color, individual phenolics and carotenoids, and in vitro antioxidant capacities. Carrot pomace presented a high content of carotenoids (84.37 ± 0.08 µg/g dw) and ash (7.78 ± 0.41% dw), providing natural orange-yellow coloration and contributing to the mineral content. Beetroot pomace recorded the highest dietary fiber (17.20 ± 0.55% dw), as well as betalains, ideal as a natural red colorant and emulsion stabilizer. Plum pomace demonstrated the strongest overall antioxidant capacity, driven by tentatively identified 3-caffeoylquinic acid (712.36 ± 0.31 μg/g dw), flavonols, and anthocyanins (264.16 ± 0.5 μg/g dw). Apple pomace provided a carbohydrate-rich matrix (77.71 ± 0.65% dw) featuring positively identified chlorogenic acid (463.16 ± 0.35 μg/g dw) and tentatively identified dihydrochalcones. These findings support the targeted valorization of these pomaces as functional food ingredients within a circular bioeconomy framework.

## 1. Introduction

According to the Food and Agriculture Organization (FAO), global apple (*Malus domestica* L.) production reached approximately 97.88 million metric tons (MMT) in 2024. China continues to lead global production, accounting for 51.29 MMT, which represents roughly 52.39% of the world’s total output. Other major international contributors include the United States of America, yielding 4.92 MMT (5.03%), and Turkey, producing 4.42 MMT (4.52%). On a regional scale, the European continent remains a major hub for apple cultivation, collectively generating 16.89 MMT, which accounts for over 17% of the global supply. Within Europe, production is largely driven by Poland (3.38 MMT), Italy (2.40 MMT), and France (1.96 MMT) [1]. Furthermore, similar robust global production volumes are observed for other raw materials utilized in the juice industry, with global yields reaching 45.15 MMT for carrots (*Daucus carota* L.) [1] and 12.69 MMT for plums (*Prunus domestica* L.) [1,2]. European production accounts for a substantial fraction of these figures as well, contributing 8.33 MMT of carrots and 2.67 MMT of plums and a total of 933.97 thousand tons for beetroot (*Beta vulgaris* L.) [3] annually. The primary European contributors to these yields include the United Kingdom (0.86 MMT) and Germany (0.85 MMT) for carrots, while Romania (0.60 MMT) and Serbia (0.39 MMT) stand out as the leading producers of plums [1]. The most significant volumes of beetroot originate from Poland (267.50 thousand tons), France (172.17 thousand tons), and Germany (168.13 thousand tons) [3]. Beetroot is also processed at an industrial scale, yielding over 177,000 tons annually in Brazil [4].

While fresh consumption, jams, and canning make up major uses of apple [5], carrot [6], plum [7], and beetroot [8], industrial juice production for these agricultural products has expanded rapidly due to growing consumer demand for functional beverages [9]. Juice extraction generates substantial amounts of side-streams—including pulp, seeds and husks—which might account for 25–30% of the initial apple mass (4 to 6 MMT globally [10]), up to 50% of carrot input [11], 35–40% of beetroot input biomass [12], and 10–60% for plum pomace and kernels [13,14,15]. This industrial process creates local waste management challenges. Currently, these residues are underutilized through land disposal, animal feed, or biochar [16,17].

These matrices are abundant in phenolic secondary plant metabolites. Specifically, anthocyanins are found in apples (cyanidin-3-glucoside, 36.1 mg/100 g fw) and plums (cyanidin-3-glucoside, 23.4 mg/100 g fw) [18], carotenoids in carrots (14.88 ± 0.30 mg/100 g dw) [19] and betalains in beetroot (as the primary antioxidant pigments) (3.39–5.19 mg/g) [4,20,21]. Together, these diverse bioactive compounds are well documented for their potent antioxidant and anti-inflammatory properties [4,18,19,22]. However, current valorization efforts predominantly focus on complex extraction steps to isolate these specific bioactives, often generating further by-products by discarding the spent biomass rich in dietary fiber and requiring specialized equipment and chemical substances [23,24]. More importantly, isolating specific compounds strips away the technological benefits of the raw material. Unlike these purified extracts, whole pomace powders retain essential techno-functional properties for food matrices. With apple pomace serving as a natural source of fiber [25] and natural sweetener thanks to its soluble sugar content [26]. Beetroot pomace can act as a natural colorant due to its red betalain pigments [12], and due to the fibrous nature of the powder it improves product emulsification [27]. Carrot and plum pomaces are also remarkable coloring agents that boost antioxidant properties of the food product [28,29], the latter can act as a preservative limiting lipid oxidation [29], all while utilizing 100% of the biomass.

Despite growing interest in agro-industrial side-streams, few studies evaluate multiple whole pomace powders, rather than extraction of bioactive compounds, under a single processing protocol. A comprehensive comparative evaluation across important European juice industry pomaces (apple, plum, carrot, and beetroot) is needed to establish and compare their composition under consistent processing conditions and potential for further applications. To overcome high moisture levels (>70%) that render these matrices prone to microbial spoilage [30], this study standardizes biomass processing through hot-air drying to halt enzymatic activity and preserve phytochemical integrity [31], followed by milling into uniform powders to increase surface area [32] and optimize the techno-functional properties in food applications.

Within the framework of circular bioeconomy, this study evaluated stabilized powders derived from pomaces resulting from industrial juice extraction. We hypothesized that stabilization into powders preserves matrix-specific bioactive signatures obtaining natural food ingredients. The main objective was to perform a comprehensive profiling of their proximal composition, color characteristics, individual phenolics, and antioxidant capacities to elucidate their technical and functional potential in novel food systems.

## 2. Materials and Methods

### 2.1. Raw Materials

Apple (AP) (*Malus domestica* Borkh., cv. Jonathan), plum (PP) (*Prunus domestica* L.) carrot (CP) (*Daucus carota* L.), and beetroot (BP) (*Beta vulgaris* L.) pomace samples were collected from a local farm in Cluj-Napoca, Romania, which produces natural juices as a secondary activity. Following collection and transport to the laboratory, the fresh by-products were immediately dehydrated. To prepare the samples for proximal and chemical characterization, the matrices were dried for 24 h at 45 °C using a commercial food dehydrator to achieve a final moisture content between 10.50 and 12.22% (Hendi Profi Line, Utrecht, The Netherlands). The drying process yielded final powder mass recovery of 18.19% for apple, 12.86% for beetroot, 13.73% for carrot, and 19.02% for plum, calculated relative to the initial fresh pomace weight. Following dehydration, the dried samples were ground into a powder using a professional laboratory mill (IKA A10, Staufen, Germany) and sieved through a sieve (0.42–0.60 mm) to obtain a fine powder, as described by our previous publication [33].

### 2.2. Chemicals and Reagents

All chemicals and solvents used in this study were of analytical or high-performance liquid chromatography (HPLC) grade. The Folin–Ciocalteu reagent, sodium carbonate (Na_2_CO_3_), aluminum chloride (AlCl_3_), sodium hydroxide (NaOH), sodium nitrite (NaNO_2_), 2,2-diphenyl-1-picrylhydrazyl (DPPH), 2,2′-azino-bis (3-ethylbenzothiazoline-6-sulfonic acid) (ABTS), potassium persulfate (K_2_S_2_O_8_), 2,4,6-tris(2-pyridyl)-s-triazine (TPTZ), ferric chloride (FeCl_3_), and sodium acetate were all purchased from Sigma-Aldrich (St. Louis, MO, USA). High-purity analytical standards of quercetin and Trolox (6-hydroxy-2,5,7,8-tetramethylchroman-2-carboxylic acid) were also obtained from Sigma-Aldrich (St. Louis, MO, USA). Solvents and reagents used for extraction and mobile phase preparation, including methanol, ethyl acetate, petroleum ether, triethylamine, glacial acetic acid, sodium chloride (NaCl), and anhydrous sodium sulfate (Na_2_SO_4_), were of analytical grade from Merck (Darmstadt, Germany). Ultrapure water was generated using a Direct-Q UV water purification system (Millipore, Burlington, MA, USA).

High-purity commercial standards used for the identification and quantification of bioactive compounds were all purchased from Sigma-Aldrich (St. Louis, MO, USA). Phenolic standards included gallic acid, rutin, catechin, and cyanidin (all ≥99% purity), as well as chlorogenic acid (≥98% purity). Carotenoid analytical standards included lycopene, lutein, and β-carotene (all ≥99% purity). For sample filtration, 0.45 µm Whatman filter papers (Cytiva, Maidstone, Kent, United Kingdom), 0.45 µm standard nylon syringe filters, and 0.45 µm Chromafil Xtra nylon syringe filters (Macherey-Nagel GmbH & Co. KG, Düren, Germany) were utilized.

### 2.3. Color and Proximate Composition

The proximate composition of the dried and milled fruit and vegetable pomace powders was determined using selected AACC approved methods [34], based on the suitability of the corresponding analytical procedures for the investigated compositional parameters. Moisture, total ash, and lipid contents were determined according to AACC 44-15.03, AACC 08-01.01, and AACC 30-25.01, respectively. Protein content was estimated from the total nitrogen content determined according to AACC 46-11.02 using a nitrogen-to-protein conversion factor of 5.7. Total fiber was determined using the AACC 32-07.01 method and total carbohydrates were calculated by subtracting crude fiber, protein, ash, lipids and moisture percentages from 100, as described by Man et al. [35].

CIELAB color coordinates, such as L* (lightness), a* (red/greed) and b* (yellow/blue) were determined using a desktop colorimeter (Shenzhen Threenh Technology Co., Ltd., Shenzhen, China), as previously described by our research group [33].

### 2.4. Phenolic Extraction Process

The extraction of polyphenols was conducted as described by Igual et al. [36]. Briefly, 0.5 g of each pomace sample was suspended in 2 mL of acidified methanol (1% HCl). The mixture was vortexed (Heidolph, Schwabach, Germany) for 1 min and subsequently subjected to ultrasound-assisted extraction for 30 min using an ultrasonic bath (Elmasonic E15H, Elma, Singen, Germany). Following centrifugation with a centrifuge Eppendorf 5804 (Eppendorf, Hamburg, Germany) (10,000 rpm, 10 min), the supernatant was collected, filtered through a 0.45 µm nylon syringe filter, and directly injected into the HPLC system to obtain the individual phenols profile and used for the determination total phenolic and flavonoid content.

#### Total Phenolic Content

Total phenolic content was determined using the Folin–Ciocalteu colorimetric assay adapted from Chis et al. [37]. For the assay, 100 μL of the extract obtained earlier was mixed with 500 μL of Folin–Ciocalteu reagent, 6 mL of distilled water, and 2 mL of 15% Na_2_CO_3_. The mixture was brought to a final volume of 10 mL with distilled water and kept in the dark at room temperature for two hours. Absorbance was recorded at 760 nm using a Shimadzu 1700 UV/visible spectrophotometer (Shimadzu Corporation, Kyoto, Japan). Quantification was performed using a gallic acid standard calibration curve (y = 1.022958x + 0.08740, R^2^ = 0.99614), with final results expressed as milligrams of gallic acid equivalents (GAEs) per gram of dry weight (mg GAE/g dw).

### 2.5. Total Flavonoid Content

Total flavonoid content (TFC) was determined using an aluminum chloride colorimetric assay, following the method outlined by Chiş et al. [37], adapted for a 96-well microplate reader (Synergy HT, BioTek Instruments, Winooski, VT, USA). Briefly, 25 μL of each methanolic extract was mixed with 100 μL of distilled water and 10 μL of 5% NaNO_2_ solution and allowed to react for 5 min. Subsequently, 15 μL of 10% AlCl_3_ solution, 50 μL of 1 M NaOH, and 50 μL of distilled water were added. Absorbance was measured at 510 nm. Quercetin was used as the reference standard, and quantification was performed using a quercetin calibration curve (R^2^ = 0.999). The results were expressed as milligrams of quercetin equivalents per gram of dry weight (mg QE/g dw).

### 2.6. Phenolic Compounds Profile

#### Phenolic Compounds Quantification

The quantification of phenolic compounds was performed using an HPLC-DAD-ESI-MS method previously described by Igual et al. [36]. An injection volume of 20 µL was utilized. The analysis was carried out on an Agilent 1200 HPLC system equipped with a quaternary pump, solvent degasser, autosampler, and a UV-Vis diode-array detector (DAD) achieved from Agilent Technologies, Santa Clara, CA, USA, coupled to an Agilent 6110 single-quadrupole mass spectrometer (Agilent Technologies, Santa Clara, CA, USA).

Chromatographic separation was achieved on a Kinetex XB-C18 column (4.6 × 150 mm, 5 µm particle size; Phenomenex, Torrance, CA, USA) maintained at 25 °C. The mobile phase consisted of (A) ultrapure water with 0.1% acetic acid, and (B) HPLC-grade acetonitrile with 0.1% acetic acid. The elution gradient (expressed as % B) was applied at a flow rate of 0.5 mL/min as follows: 0–2 min, 5% B; 2–18 min, 5–40% B; 18–20 min, 40–90% B; 20–24 min, 90% B; 24–25 min, 90–5% B; 25–30 min, 5% B.

Spectral data were recorded over 200–600 nm for all peaks, and chromatograms were monitored at λ = 280, 340, 480, and 536 nm. Mass spectrometry (MS) was operated in positive electrospray ionization (ESI+) full-scan mode under the following conditions: capillary voltage 3000 V, drying gas temperature 350 °C, nitrogen flow rate 7 L/min, and an *m*/*z* range of 120–1200. Data acquisition and interpretation were performed using Agilent ChemStation software (Rev B.02.01-SR2).

Phenolic compounds were identified based on their chromatographic and spectral characteristics, including retention time (RT), UV–Vis absorption maxima (λmax), and mass-to-charge ratio (*m*/*z*). Gallic acid, chlorogenic acid, rutin, cyanidin, and catechin were positively identified by direct comparison of their RT, UV–Vis, and mass spectral characteristics with those of the corresponding authentic standards analyzed under the same chromatographic conditions. All other phenolic compounds, for which authentic standards were not available, were tentatively identified based on the combined evaluation of their RT, UV–Vis absorption characteristics, and *m*/*z* values, with assignments supported by comparison with data reported in the Database on Polyphenol Content in Foods [38] and the relevant literature. The experimental RT, λmax, and *m*/*z* values used for compound identification are also reported.

Quantification was performed using external calibration curves prepared from the available commercial standards. Hydroxybenzoic acids were quantified as gallic acid equivalents (R^2^ = 0.9978; LOD = 0.35 µg/mL; LOQ = 1.05 µg/mL), hydroxycinnamic acids as chlorogenic acid equivalents (R^2^ = 0.9937; LOD = 0.41 µg/mL; LOQ = 1.64 µg/mL), flavonols as rutin equivalents (R^2^ = 0.9981; LOD = 0.21 µg/mL; LOQ = 0.84 µg/mL), anthocyanins and betacyanins as cyanidin equivalents (R^2^ = 0.9951; LOD = 0.36 µg/mL; LOQ = 1.44 µg/mL), and flavanols as catechin equivalents (R^2^ = 0.9985; LOD = 0.18 µg/mL; LOQ = 0.72 µg/mL).

### 2.7. Carotenoids Profile

#### 2.7.1. Extraction Process

The extraction of carotenoids was conducted as described by Szabo et al. [39]—1 g of each sample was extracted with 5 mL of a methanol/ethyl acetate/petroleum ether mixture (1:1:1, *v*/*v*/*v*) via sonication for 15 min in an ultrasonic bath (Elmasonic E15H, Elma, Singen, Germany) and centrifugation (10,000 rpm, 10 min), using an Eppendorf 5804 centrifuge (Eppendorf, Hamburg, Germany). The washing steps were repeated until the sample pellet was completely decolorized. The pooled organic supernatants were then washed with 15% NaCl, dried, and evaporated to dryness under reduced pressure using a rotary evaporator (Hei-VAP Expert, Heidolph Instruments GmbH & Co. KG, Schwabach, Germany). Finally, the dried residue was redissolved in 5 mL of ethyl acetate, filtered, and prepared for HPLC injection.

#### 2.7.2. Carotenoids Quantification

The targeted analysis of carotenoids was conducted following the methodology described by Szabo et al. [39], using an Agilent 1200 HPLC system (Agilent Technologies, CA, Santa Clara, USA) equipped with a UV-Vis diode-array detector (DAD). Separation was achieved using a Nucleodur 300-5 C18 column (250 × 4.6 mm, 5 µm; Macherey-Nagel, Düren, Germany) maintained at 25 °C. The mobile phases consisted of acetonitrile: water (9:1, *v*/*v*) with 0.25% triethylamine (A) and ethyl acetate with 0.25% triethylamine (B). The gradient started at 90% A at 0 min, decreased to 50% A at 10 min, then to 10% A at 20 min. The flow rate was 1 mL/min, and the chromatograms were registered at λ = 450 nm. Data acquisition was managed using the Agilent ChemStation software (Rev B.02.01-SR2). Carotenoids were identified and quantified using calibration curves of commercial analytical standards of lutein (R^2^ = 0.9945) and β-carotene (R^2^ = 0.9931).

### 2.8. Antioxidant CapacityAnalysis

Prior to the analyses, the pomace samples were mixed with 95% methanol and vortexed (Heidolph, Schwabach, Germany) for 3 min. The samples were then subjected to ultrasound-assisted extraction (Elmasonic E15H, Elma, Singen, Germany) for 10 min at room temperature. Following extraction, the mixture was centrifuged (Eppendorf 5804, Eppendorf, Hamburg, Germany) at 1000 rpm for 10 min at 4 °C; the supernatant was collected for the antioxidant assays.

The in vitro antioxidant capacity of the pomace extracts was evaluated using three methods: first, by measuring their free radical scavenging activity against the stable 2,2-diphenyl-1-picrylhydrazyl (DPPH) radical, according to the analytical protocol described by Agudelo et al. [40]. The spectrophotometric measurements of the reaction mixtures were recorded at 515 nm using a Helios Zeta UV-visible spectrophotometer (Thermo Electron Corporation, Loughborough, UK). A calibration curve was prepared with Trolox at concentrations of 50 to 350 ppm, R^2^ = 0.9996. The final free radical scavenging results were expressed as milligrams of Trolox equivalents (TE) per 100 g of sample (mg TE/100 g).

Second, the ABTS+ radical-cation scavenging capacity of the pomace extracts was evaluated following the protocols established by Crişan et al. [41], with minor modifications. Briefly, a 10 μL aliquot of each sample extract was combined with 231 μL of a 7 mM ABTS+ working solution in a microplate well. The reaction mixture was incubated for 30 min. Following the incubation period, absorbance was measured with a Spectra-Max ABS Plus microplate reader (Molecular Devices, San José, CA, USA). Quantification was performed using a Trolox standard calibration curve prepared over a concentration range of 50 to 500 ppm (R^2^ = 0.9994) and the results were expressed as in the DPPH assay.

Finally, the reducing capacity of the extracts was determined following the method outlined by Ferreira et al. [42]. Briefly, 35 μL of diluted sample (1:10) was mixed with 265 µL of freshly prepared FRAP reagent (comprising 0.3 M acetate buffer, 10 mM TPTZ, and 20 mM ferric chloride in a 10:1:1 ratio). Following a 30 min incubation at 37 °C, absorbance was recorded at 595 nm using the same microplate reader as previously used. Reducing power was quantified using a Trolox calibration curve (25–500 µM; R^2^ = 0.9997) and expressed similarly to the DPPH assay.

### 2.9. Statistical Analysis

Statistical analyses were performed using IBM SPSS Statistics 26 (IBM Corp., Armonk, NY, USA). Data normality and homogeneity of variances were assessed using the Shapiro–Wilk and Levene’s tests, respectively. Differences among samples were evaluated by one-way ANOVA followed by Tukey’s HSD test (*p* < 0.05). Results are expressed as mean ± SD (*n* = 3). Pearson’s correlation analysis was applied to evaluate relationships among phytochemical and antioxidant parameters. Principal Component Analysis (PCA) was performed using GraphPad Prism 9.3.0 (GraphPad Software, Boston, MA, USA) to visualize patterns and relationships within the dataset.

## 3. Results

### 3.1. Color Profile and Proximate Composition Analysis

The proximate composition and color parameters of the dried and milled BP, CP, PP, and AP are presented in Table 1.

Dehydration is an important step in the stabilization of fruit and vegetable by-products. In the present study, the moisture content of the dried pomaces ranged from 10.50 ± 0.03% in AP to 12.22 ± 0.05% in BP, remaining below the 15% threshold associated with microbiological stability [43]. Lower moisture contents have been reported in previous studies. Antonic et al. [44] reported values ranging from 3.97 to 9.75% for apple pomace, whereas Sahni et al. [45] reported moisture contents of 6.82 ± 0.03% and 7.60 ± 0.05% for beetroot and carrot pomace powders, respectively.

The comparatively higher residual moisture observed in the present study may be partly attributed to the lower drying temperature applied (45 °C), as increasing drying temperature has been shown to enhance moisture removal in apple pomace. Moreover, differences in final moisture content among apple cultivars subjected to identical drying conditions indicate that raw-material characteristics may also contribute to the variability reported across studies [45,46].

A clear compositional divide was observed between the fruit pomaces and root/vegetable. CP and BP were characterized by significantly higher (*p* < 0.05) protein contents (9.12 ± 0.05% dw and 7.75 ± 0.21% dw, respectively) compared to fruit pomaces. According to the literature, the higher protein content of BP may be associated with its nitrogen-containing betalains [45]. Sahni et al. [45] reported 15.85 ± 0.03% protein in beetroot pomace powder, compared with 4.59 ± 0.04% and 6.12 ± 0.03% in apple and carrot pomaces, respectively. Antonic et al. [44] reported moisture contents of 3.97–9.75% for apple pomace, lower than the 10.50 ± 0.03% obtained for AP in the present study. Such differences may be related to the drying conditions, as Milala et al. [47] demonstrated that the drying method influences the characteristics of pomace powders.

Ash content, which reflects total mineral density, was significantly higher (*p* < 0.05) in the root pomaces than in the fruit pomaces, peaking in CP (7.78 ± 0.41% dw) and surpassing the ash range of 5.29–5.89% dw reported by Luca et al. [48], followed by BP (5.45 ± 0.03% dw), almost matching the literature reported value of 5.62% [49]. This suggests that root pomaces hold potential for use in mineral and protein fortifications of foods.

The fruit pomaces exhibited higher carbohydrate content, with AP (77.71 ± 0.65% dw) and PP (75.11 ± 0.91% dw) recording the highest values, making AP and PP suitable for integration into bakery, dairy and confectionery food products [25]. Meanwhile BP emerged as a source of total fiber (17.20 ± 0.55% dw), positioning it as a functional ingredient for fortifying breads, pastas and snacks [12]. Additionally, due to fiber’s high water-holding capacity, BP has strong potential for applications in meat products to improve moisture retention and emulsion stability and serve as a fat replacer [12,50]. According to the literature, the drying process used has an important role in the fiber content of plum powder, Milala et al. [47].

As expected, lipid content was low across all matrices, ranging from 0.12 ± 0.03% dw in BP to 1.28 ± 0.06% dw in CP. Higher and relatively variable lipid contents have been reported in the literature. Antonic et al. [44] reported values ranging from 0.26 to 8.49% for apple pomace, while Sahni et al. [45] reported lipid contents of 3.48% and 1.44% for carrot and beetroot pomace powders, respectively. These differences may reflect variations in raw material composition and processing conditions among studies.

From an application perspective, the compositional differences among the investigated pomaces suggest distinct valorization routes. Their high dietary fiber content supports their use as fiber-enriching ingredients in functional food formulations, whereas phenolic-rich pomaces could serve as sources of natural antioxidants. Moreover, pomaces with high concentrations of anthocyanins, betacyanins, or carotenoids may have additional potential as sources of natural colorants or as raw materials for the targeted recovery of these bioactive compounds [51,52].

However, the successful incorporation of these pomace powders into food matrices requires dealing with technological and sensory trade-offs. High dietary fiber levels in BP and AP alter water absorption kinetics in bakery applications, which can disrupt gluten-network formation and reduce product volume if substitution rates exceed 5–10% [53]. Furthermore, high polyphenol concentrations in PP and AP can impart sensory astringency and bitterness [26,54], whereas geosmin present in BP introduces earthy off-flavors to sensitive formulations [55]. From a physiological standpoint, cell-wall-bound phenolics require colonic microbial fermentation for enzymatic release, making their systemic bioavailability dependent on gut microbiota composition [56].

Color is one of the important factor in consumer acceptance [57] and in these pomace powders, it hints at the preservation of functional pigments. The low lightness values of BP (L* = 2.58 ± 0.04) and PP (L* = 2.54 ± 0.08) reflect high pigment retention. Specifically, BP exhibited a vibrant red value (a* = 88.85 ± 0.07), while PP demonstrated a red-purple profile (a* = 60.38 ± 0.23), these profiles make BP and PP potential alternatives for synthetic red coloring agents linked to adverse effects on human health [58,59].

CP was represented by the brightest color profile (L* = 49.02 ± 0.05) with a yellow, red hue (b* = 35.86 ± 0.13, a* = 12.08 ± 0.56), specific for β-carotene accumulation [6]. This allows CP to act as a natural orange-yellow colorant for snacks, pasta, and bakery products [6].

### 3.2. Total Phenols and Total Flavonoid Content

Significant differences in total phenolic content were observed among the analyzed pomace powders (*p* < 0.05). PP exhibited the highest value (183.23 ± 0.45 mg GAE/100 g dw), followed by AP with a value of 148.52 ± 0.88 mg GAE/100 g dw, whereas CP and BP showed lower phenolic concentrations (98.78 ± 0.21 and 83.45 ± 0.13 mg GAE/100 g dw, respectively). Higher TPC values have been reported for apple pomace, with Gorjanović et al. [60] reporting values ranging from 460 to 810 mg GAE/100 g. In contrast, Tumbas Šaponjac et al. [61] reported a TPC of 89.06 ± 4.62 mg GAE/100 g dw for beetroot pomace, which was comparable to that obtained in the present study. For carrot processing waste, Bas-Bellver et al. [62] reported a TPC of 203 mg GAE/100 g dw, exceeding the value determined for CP in the present study.

According to the literature, the phenolic content of fruit and vegetable pomaces may be influenced by several factors, including cultivar, juice-processing technology, subsequent processing, and storage conditions. In addition, the retention of phenolic compounds in dried pomaces is strongly dependent on the drying conditions applied [60,61].

With respect to total flavonoid content, the same trend was observed among samples. PP exhibited the highest value (5.52 ± 0.22 QE/g dw), followed by AP, with a value of 4.50 ± 0.08 QE/g dw, 3.61 ± 0.12 QE/g dw and 2.12 ± 0.08 QE/g for CP and BP samples, respectively. For apple pomace, Gurev et al. [63] reported a lower TFC value of 2.13 ± 0.13 mg QE/g DW, whereas Gorjanović et al. [60] reported considerably higher values ranging from 18.6 to 34.6 mg QE/g. For beetroot pomace, Gorjanović et al. [64] reported a TFC of 1.43 ± 0.10 mg QE/g, which was slightly lower than that obtained for BP in the present study. The variability among studies may be attributed to differences in raw material characteristics, processing conditions, and extraction procedures.

The higher total phenolic and flavonoid contents observed in PP and AP samples compared with CP and BP samples may be attributed to the species-specific accumulation of polyphenols in fruit tissues, particularly in peel-rich fractions that remain concentrated after juice extraction [65,66]. In contrast, the lower values recorded for carrot and beetroot pomaces likely reflect differences in the distribution and retention of bioactive compounds within the plant matrix during processing [67,68]. Moreover, the retention of phenolic compounds may be enhanced by thermal treatments that inactivate polyphenol oxidase, an enzyme responsible for the oxidation and degradation of phenolics during fruit and vegetable processing [68].

The retention of thermolabile bioactives across all pomaces is directly attributable to the low-temperature dehydration (45 °C for 24 h) applied. Thermal stabilization at 45 °C reduced moisture below 12%, inhibiting polyphenol oxidase (PPO) and peroxidase enzymatic degradation [69] without triggering the thermal ring-cleavage of anthocyanins observed at temperatures > 60 °C [70]. Furthermore, milling and sieving to a controlled particle size (0.42–0.60 mm) mechanically disrupted outer cell wall barriers, expanding the specific surface area and improving solvent mass transfer during analytical extraction.

### 3.3. Carotenoid and Phenolic Profile

The quantification of carotenoid profiles (Table 2) highlighted CP as the most abundant carotenoid matrix, recording a total carotenoid content of 84.37 ± 0.08 µg/g dw, from which the main amounts were represented by β-carotene (61.593 ± 0.07 μg/g dw) and lutein (22.780 ± 0.05 μg/g dw). Comparing this β-carotene yield to the 82.85 μg/g baseline typical of fresh carrots [6] underscores the retention in the matrix after juicing. This aligns with the findings of Moulick et al. that carrot tissues naturally accumulate carotenoids compared to other root vegetables [71].

On the other side, the PP carotenoid amount was lower compared with the carrot and exhibited a value of 32.83 µg/g dw, from which β-carotene and lutein were identified with values of 21.262 ± 0.35 μg/g dw of β-carotene and 11.571 ± 0.74 μg/g dw of lutein, whereas AP retained lower amounts (5.245 ± 0.31 μg/g dw and 1.145 ± 0.12 μg/g dw, respectively). Finally, BP recorded the lowest overall carotenoid concentrations. Lutein was entirely absent in the beetroot matrix, and β-carotene was detected only in minor quantities (3.286 ± 0.23 μg/g dw), a minimal lipophilic profile that is consistent with the total carotene baseline reported for whole dried beetroot powders (5.94 μg/g) [71].

With respect to the samples phenolic profile, a total number of 28 individual compounds were identified (Table 2), of which four were positively identified using authentic standards and 24 were tentatively identified based on chromatographic and spectral data. In the PP sample, the main amounts identified chromatographically were represented by hydroxycinnamic acids, specifically 3-caffeoylquinic acid (Rt 9.98 min) reaching a concentration of 712.36 ± 0.31 µg/g dw, and flavonols such as quercetin-rutinoside (Rt 15.51 min) at 309.78 ± 0.31 µg/g dw. The anthocyanin fraction co-elutes with other phenolics in the 11 min region of the 280 nm trace and was represented primarily by cyanidin-rutinoside/glucoside (Rt 11.16–11.98 min) with a combined value of 161.70 ± 0.28 µg/g dw. These concentrations fall within the general ranges reported by Michalska et al. [72] for PP powders. Specifically, 3-caffeoylquinic acid fell midway between their thermally dried (384–967 µg/g dw) values, quercetin-rutinoside closely matched their observed range (296–474 µg/g dw), and total cyanidin derivatives were higher than in all thermally processed samples (22–128 µg/g dw).

CP and AP samples highlighted similar total phenolic loads, recording 22.25 ± 0.36 and 22.78 ± 0.05 mg GAE/g dw, respectively, with the CP value falling within the 19.55–24.05 mg GAE/g range established by Luca et al. [73]. In the CP sample, the main amounts were represented by chlorogenic and 3-feruloylquinic acids, with values of 217.78 ± 0.34 and 148.90 ± 0.37 µg/g dw, respectively, along with 2,3-dihydroxybenzoic acid (157.08 ± 0.32 µg/g dw) and gallic acid (52.24 ± 0.52 µg/g). Conversely, the AP profile was mainly characterized by chlorogenic acid (463.16 ± 0.35 µg/g dw), and quercetin-arabinoside (352.29 ± 0.18 µg/g dw). Notably, both concentrations align perfectly with the baseline literature ranges of 405–2299 mg/kg dw and 27–579 mg/kg dw, respectively, as highlighted by Ziobro et al. [74], combined with high levels of dihydrochalcones, represented by phloretin-xylosyl-glucoside (84.81 ± 0.12 µg/g dw).

The BP sample presented a lower phenolic baseline, aligning with previous research (12.96 mg GAE/g) [71], and exhibited a value of 13.05 ± 0.19 mg GAE/g dw. Its individual phenolic profile lacked complex flavonols and was almost entirely defined by simple hydroxybenzoic acids, from which only protocatechuic acid and 3-hydroxybenzoic acid were identified as major components with values of 142.80 ± 0.32 and 69.79 ± 0.12 µg/g dw, respectively. Such differences in the phenolic profiles of pomace may also be partly related to the distribution of phenolic compounds between the liquid and solid fractions during juice processing. In the case of juice pressing without the aid of chemical or enzymatic treatments to cleave the bonds between polysaccharides (cellulose, hemicellulose, pectin) and phenolics, the mechanical breakdown of cellular vacuoles releases highly hydrophilic, unbound compounds—primarily monomeric anthocyanins (e.g., cyanidin-3-glucoside, cyanidin-3-rutinoside) and soluble caffeoylquinic acids—directly into the juice [75]. This can occur because hydroxybenzoic acids (such as 2,3-dihydroxybenzoic and p-hydroxybenzoic acids), lipophilic pigments, and high-molecular-weight proanthocyanidins are bound via ester, ether, or hydrophobic bonds to structural polysaccharides (cellulose, hemicellulose, pectin) and lignin [76]. Consequently, the solid fraction remaining after juice extraction may retain a substantial proportion of matrix-associated and bound phenolic compounds together with dietary fiber, further supporting the potential valorization of pomace as a source of functional ingredients [63,76]. However, since free and bound phenolic fractions were not separately quantified in the present study, their relative contribution to the phenolic profile of the investigated pomaces cannot be established. Further studies involving the separate characterization of free and bound fractions would therefore provide a more comprehensive assessment of their bioactive potential.

### 3.4. Antioxidant Capacity

**Figure 1:**
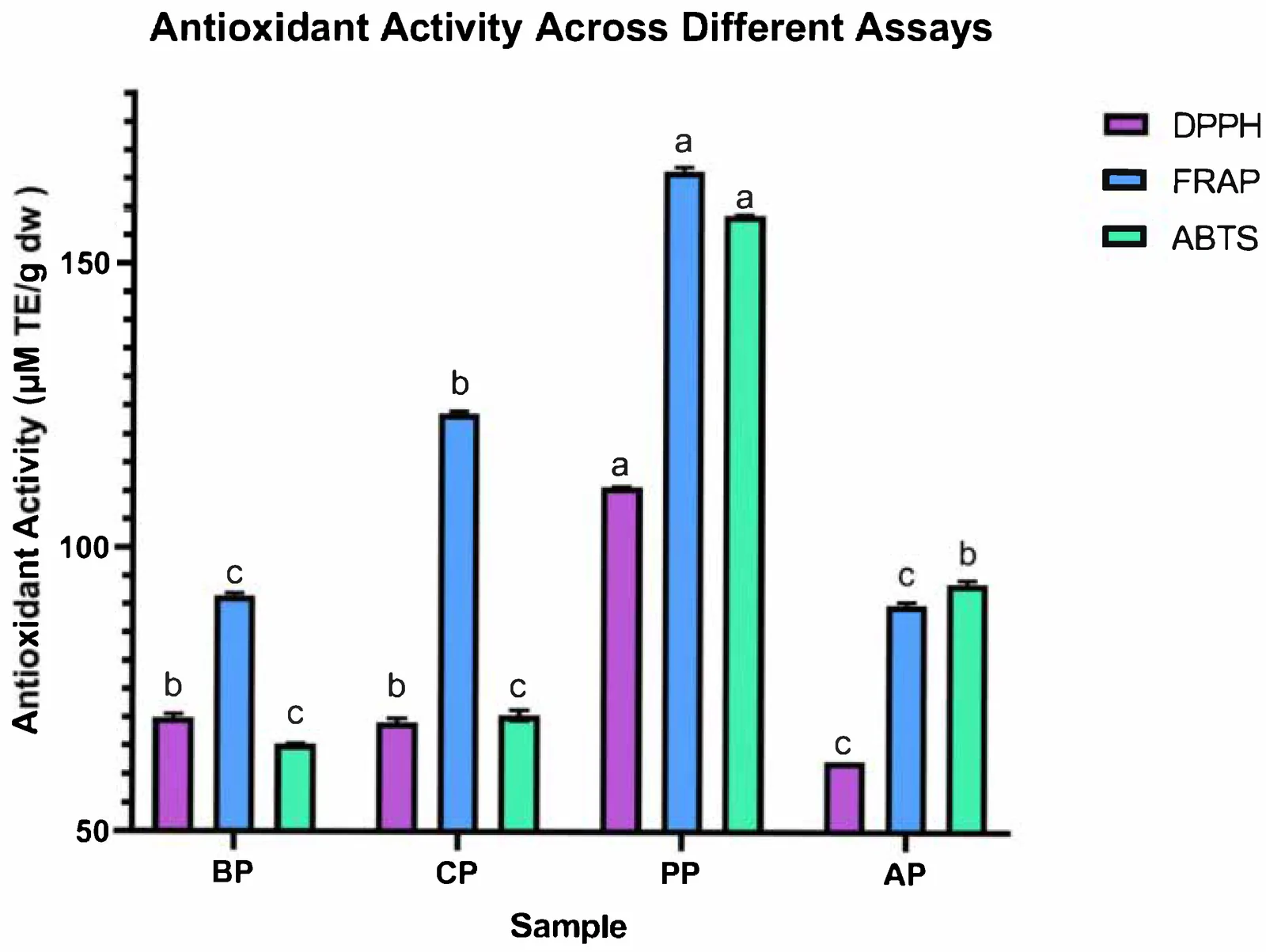
Antioxidant capacities of pomace samples evaluated via DPPH, FRAP, and ABTS assays. Results are expressed as µM TE/g dw. Values represent the mean ± standard deviation. Different lowercase letters indicate statistically significant differences among samples for each respective assay (*p* < 0.05). BP, Beetroot Pomace; CP, Carrot Pomace; PP, Plum Pomace; AP, Apple Pomace; TE, Trolox Equivalents; dw, dry weight.

The in vitro antioxidant capacity of the pomaces (Figure 1), evaluated using complementary radical-scavenging (DPPH, ABTS) and reducing-power (FRAP) assays, aligned with their established phytochemical profiles. PP demonstrated the highest antioxidant potential across all three mechanisms by a significant margin, yielding 110.89 ± 0.08 μM TE/g dw for DPPH, 158.69 ± 0.13 μM TE/g dw for ABTS, and 166.47 ± 0.71 μM TE/g dw for FRAP.

CP and AP displayed lower antioxidant activities compared to the antioxidant activity of PP. CP recorded 123.57 ± 0.47 μM TE/g dw in the FRAP assay and a DPPH scavenging value of 69.17 ± 0.70 μM TE/g dw. This may be attributed to spectral interferences caused by the high carotenoid content of the CP. As noted by Arnao [77], naturally colored compounds such as carotenoids can interfere with absorbance readings in the visible region, particularly at the 515 nm wavelength used for DPPH, leading to an underestimation of the actual radical-scavenging activity. This trend is consistent with recent studies on CP, in which ferric-reducing power (FRAP) has been shown to be three to four times higher than radical-scavenging activity [78]. Conversely, AP showed an ABTS radical scavenging of 93.80 ± 0.66 μM TE/g dw compared to carrot’s 70.49 ± 0.94 μM TE/g dw. BP displayed antioxidant capacities across the assays were: DPPH: 69.96 ± 0.66 μM TE/g dw; ABTS: 65.33 ± 0.07 μM TE/g dw; FRAP: 91.52 ± 0.47 μM TE/g dw.

### 3.5. Correlation Between Phytochemical Profiles and Antioxidant Capacity

**Figure 2:**
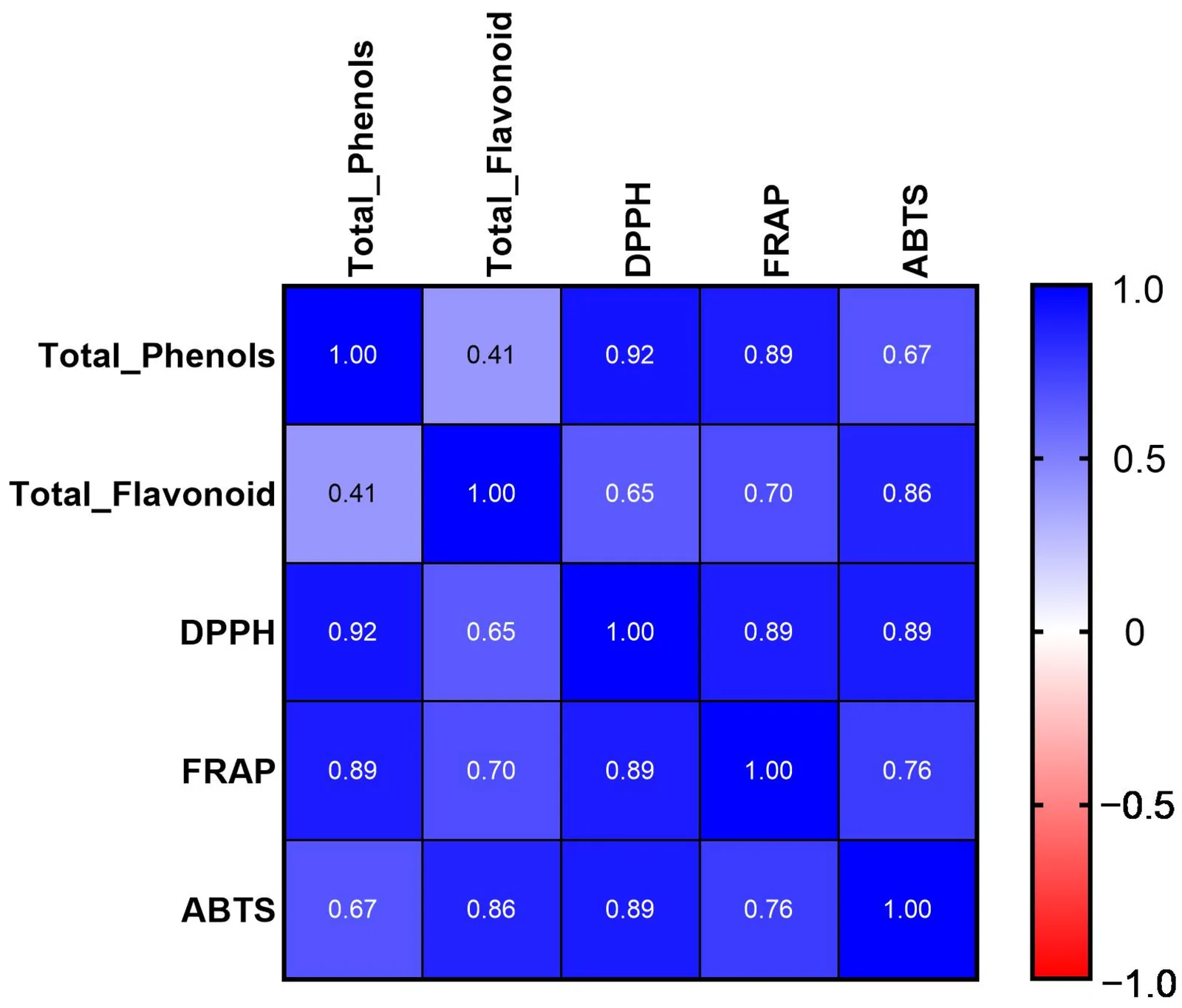
Pearson correlation matrix showing exploratory associations among total phenolic content (TPC), total flavonoid content (TFC), and antioxidant responses determined by DPPH, FRAP, and ABTS assays in the investigated pomaces. Correlation coefficients are presented descriptively and should be interpreted cautiously given the limited number of distinct plant matrices analyzed.

To further explore the relationships between the phytochemical parameters and antioxidant responses of the four investigated pomaces, Pearson correlation analysis was performed (Figure 2). Given the limited number of distinct plant matrices included in the analysis, the resulting correlation coefficients were interpreted descriptively as exploratory associations within the present dataset rather than as evidence of generalizable relationships.

The correlation analysis showed different degrees of association between the phytochemical parameters and the antioxidant assays. TPC showed strong positive associations with DPPH (r = 0.92) and FRAP (r = 0.89), whereas its association with ABTS was lower (r = 0.67). TFC showed its strongest association with ABTS (r = 0.86), followed by FRAP (r = 0.70) and DPPH (r = 0.65). The association between TPC and TFC was comparatively lower (r = 0.41). Among the antioxidant assays, DPPH showed strong positive associations with both FRAP and ABTS (r = 0.89), whereas the association between FRAP and ABTS was lower (r = 0.76). These differences indicate that the relationships between phytochemical parameters and antioxidant responses were not uniform across the assays and should therefore be interpreted cautiously.

The different patterns observed among ABTS, FRAP, and DPPH may be explained, at least in part, by differences in their underlying reaction mechanisms and assay conditions. The chemical structure of phenolic compounds can influence their reactivity toward different radicals. Platzer et al. [79] reported that steric effects associated with certain flavanols and dihydrochalcones can limit their reaction with the DPPH radical, whereas the ABTS radical cation is less affected by such steric constraints. Differences in the reaction medium may also influence assay responses. As described by Arnao [77], ABTS can be applied in both aqueous and organic systems and can therefore respond to both hydrophilic and lipophilic antioxidants, whereas DPPH is predominantly used in organic media.

Differences involving FRAP may additionally reflect its distinct analytical mechanism. FRAP primarily measures ferric-reducing capacity through a single-electron transfer (SET) mechanism, whereas ABTS and DPPH may involve electron- and hydrogen-transfer processes depending on the experimental conditions [80]. Consequently, chemically heterogeneous pomace matrices may produce different relative responses across the antioxidant assays. Thus, the ABTS, DPPH, and FRAP results should be considered complementary measures of in vitro antioxidant properties rather than interchangeable indicators expected to produce identical patterns. In view of the limited number of matrices investigated, the correlations shown in Figure 2 are presented as exploratory associations and do not support general conclusions regarding relationships between individual phytochemical parameters and antioxidant capacity.

**Figure 3:**
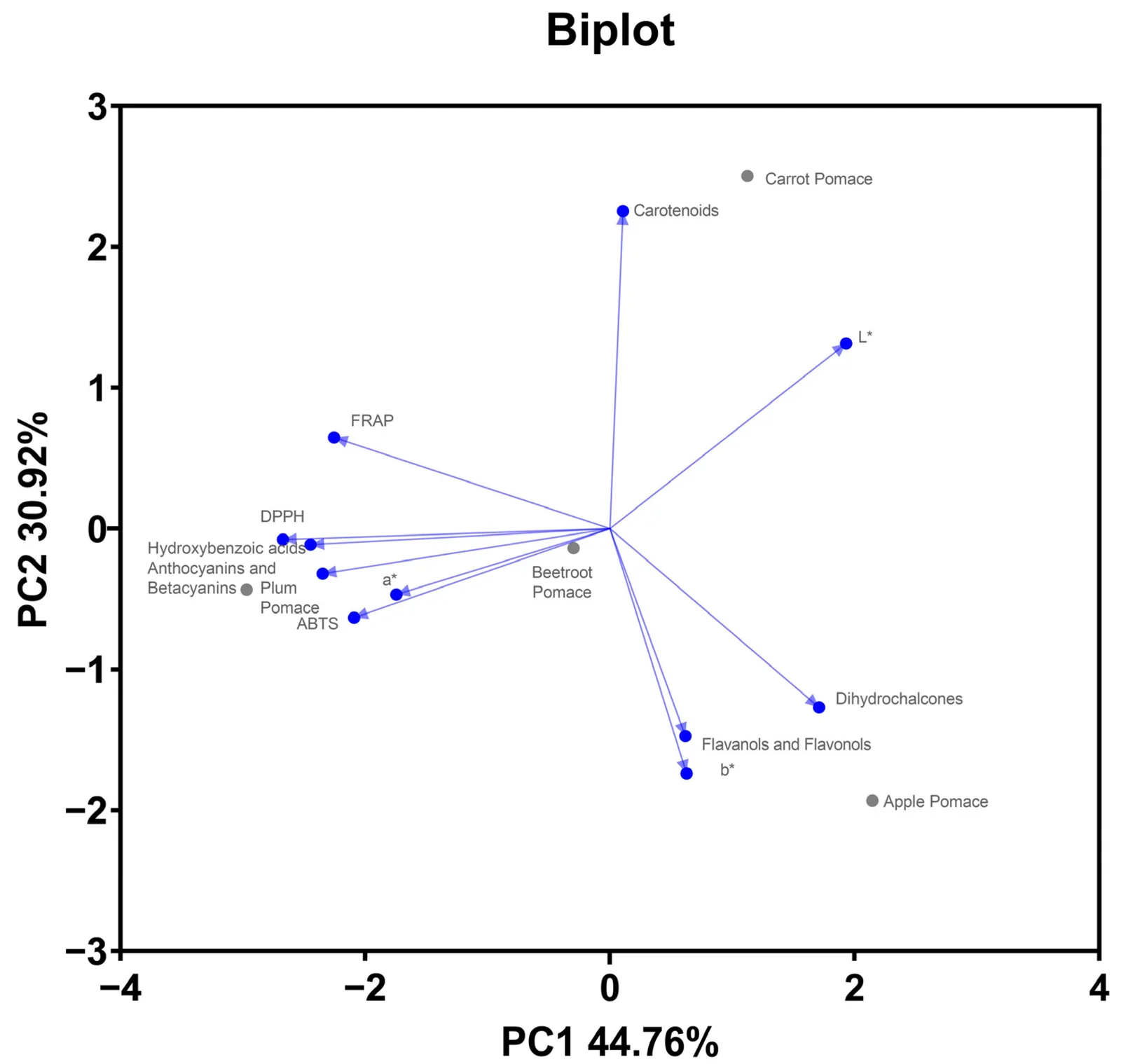
Principal Component Analysis (PCA) biplot displaying the multivariate relationships among bioactive compound groups (hydroxybenzoic acids, flavonols and flavanols, anthocyanins and betacyanins, dihydrochalcones, and carotenoids), antioxidant activities (DPPH, FRAP, ABTS), and CIELAB color parameters (L*, a*, b*) across four pomace matrices (apple, beetroot, carrot, and plum). The first two principal components account for 75.68% of the total variance (PC1 = 44.76%, PC2 = 30.92%). Data were standardized prior to analysis, and principal components were selected based on the Kaiser criterion (eigenvalues > 1). Blue vectors denote variable loadings, while gray points represent score positions for each pomace sample.

To evaluate the interrelationships between color attributes, bioactive composition, and antioxidant capacity across the different agro-industrial pomace sources, a Principal Component Analysis (PCA) biplot was constructed (Figure 3). The first two principal components explained 75.68% of the total variance, with PC1 accounting for 44.76% and PC2 for 30.92%, demonstrating that the selected parameters effectively discriminate among the four byproduct matrices.

The first principal component (PC1, horizontal axis) accounted for 44.76% variance and serves as a benchmark for antioxidant activity and phenolic load. This axis separates the most active matrices, most notably PP located on the far left, from the less active matrices (AP, CP, and BP) positioned to the right. The second principal component (PC2, vertical axis) accounted for 30.92% variance and represents a botanical, pigment and chemical class discriminator. It separates CP, rich in carotenoids and characterized by lightness (L*), situated in the upper quadrant, from AP, rich in specific flavonoid sub-classes and yellow hues (b*), situated in the lower quadrant.

PP is positioned on the far left of PC1, perfectly aligned with the vectors of antioxidant assays, as well as hydroxybenzoic acids, anthocyanins and betacyanins, and the redness parameter (a*). This arrangement indicates that hydroxybenzoic acids drive the antioxidant activity and the physical color observed in the plum matrix. Individual phenolic profile by HPLC supports this, revealing the presence of compounds in the hydroxybenzoic acid subclass, specifically 2,3-dihydroxybenzoic acid (191.73 ± 0.23 μg/g dw), 3-caffeoylquinic acid (712.36 ± 0.31 μg/g dw), and 5-caffeoylquinic acid (200.74 ± 0.52 μg/g dw). These values are comparable to those reported in the case of vacuum-dried plums (625,9 ± 25,8 μg/g dw for 3-Caffeoylquinic acid and 242,9 ± 15,1 μg/g dw) [81]. Furthermore, these values found in the PP parallel findings in the broader literature concerning stone fruit (*Prunus* genus) waste. Research confirms that the side-stream from processing stone fruits retains p-hydroxybenzoic and chlorogenic acid [7].

AP maps in the bottom right quadrant of the biplot, driven by vectors of dihydrochalcones and flavonols and flavanols, as well as the yellowness parameter (b*). The spatial alignment between these phenolic classes and b* highlights that yellow-brown flavonoids are the primary contributors to the chromaticity of this matrix. The specific chemical composition of this matrix is characterized by 5-caffeoylquinic acid (463.16 ± 0.35 μg/g dw) and dihydrochalcones; phloridzin (13.28 ± 0.25 μg/g dw), a marker of the AP [82]; and phloretin-xylosyl-glucoside (84.81 ± 0.12 μg/g dw). The obtained value of 5-caffeoylquinic acid exceeds the values reported by Hobbi et al. [83] on-peel fraction (263.7 ± 11.3 μg/g dw) and pulp and peel (170.5 ± 4.6 μg/g dw). A reason might be that AP also contains core tissues, where high concentrations of chlorogenic acid are reported (970.5 ± 24.5 μg/g dw) [83].

Beyond anatomical tissue distribution, these compositional profiles can be further related to the juice pressing process, during whic a considerable proportion of soluble compounds is transferred into the juice fraction [84]; insoluble and cell-wall-bound components remain retained in the pomace [85]. Soluble, low-molecular-weight compounds, similar to PP, hydrophilic hydroxycinnamic acids (such 5-caffeoylquinic acid) and monomeric flavan-3-ols (such as (+)-catechin and (−)-epicatechin), are partitioned into the juice matrix [84]. Conversely, components associated with the cell walls remain in the pomace [86]. Specifically, hydrophobic flavonols (predominantly quercetin glycosides), high-molecular-weight polymeric proanthocyanidins (condensed tannins complexed with cell-wall polysaccharides like pectin and cellulose), and dihydrochalcones (including phloridzin and phloretin glycosides characteristic of peel matrices) are concentrated in the solid fraction [87].

BP occupies a central position near the origin of the biplot, moderately toward the left alongside the vector representing anthocyanins, betacyanins and a*. While it shares this pigment class with PP along PC1, it lacks the phenolic acid load that pushes plum further left. This positioning confirms that the BP matrix is chemically characterized by nitrogenous betalain pigments, specifically betacyanins. Studies on the betacyanin content of beetroot juice and sun-dried powder reveal values of 2810 ± 100 μg/g dw [88] and 626.7 ± 4.6 μg/g dw [8], respectively—higher values than those observed in our study (281.57 μg/g dw). The lower betacyanin concentration observed in our BP compared to the literature values for beetroot juice and dried powder may be attributed to the hydrosoluble nature of the pigments [89].

During mechanical juicing, the majority of the betacyanin-rich fraction is partitioned into the juice. The remaining pomace mainly consists of fibers, where the residual pigments are not accessible. This dynamic may be explained by the relationship between the free (soluble) and bound fractions of phenolic compounds [90]. During juice production, a considerable proportion of soluble compounds gets transferred into the liquid phase, whereas insoluble-bound components are left behind in the pomace matrix [91].

On the other hand, CP is represented in the upper-right quadrant of the PCA biplot, drawn almost entirely by the vectors for carotenoids and lightness (L*). It remains isolated from the vectors of FRAP, DPPH, and ABTS, as well as from the hydrophilic phenolic groupings on the opposite side of the plot. The alignment with L* reflects the higher visual brightness and light-reflecting properties of the carrot matrix compared to the darker, pigmented plum and beetroot matrices. Furthermore, its separation along PC2 underscores the distinct lipophilic nature of carotenoid pigments [92] compared to the water-soluble phenolics driving the antioxidant activity in the other matrices. Unlike other matrices, despite this distinct spatial separation from the major antioxidant clusters, the analyzed CP demonstrates a complete array of hydroxybenzoic acids, consistent with findings of Ziobro et al. [78]. This clustering can be justified by the fact that mechanical juice extraction primarily removes the aqueous phase along with soluble compounds [48], leaving a solid residue concentrated with insoluble fibers [93]. Because lipophilic carotenoids like β-carotene and α-carotene are inherently water-insoluble and physically trapped within the hydrophobic protein–lipid matrices of intact cellular chromoplasts [94], they naturally accumulate into the remaining solids.

Consequently, the pomaces analyzed in this study hold potential as concentrated sources of bound phenolic compounds and dietary fiber [95]. Understanding this specific distribution of bioactive compounds between soluble and bound fractions can provide a much stronger scientific rationale for the proposed valorization of these materials, repositioning them from processing waste to functional ingredients [96].

From an industrial scalability perspective, upcycling these materials is economically viable because pomaces are generated at centralized processing facilities, eliminating farm-to-factory logistic needs. Furthermore, converting pomaces directly into powders bypasses the need for complex extraction processes and leaves no secondary residues behind, ensuring total resource utilization [97]. Convective air-drying at 45 °C represents the energy and cost-efficient compromise compared to freeze-drying [98]. Nevertheless, seasonal supply fluctuations and high powder hygroscopicity mandate moisture-barrier storage solutions to prevent caking and oxidative degradation over extended shelf lives [99].

## 4. Conclusions and Perspectives

The investigated pomaces exhibited distinct compositional and phytochemical profiles, supporting their differentiated potential as food ingredients. Beetroot pomace, with the highest fiber content and characteristic betalain pigmentation, may be suitable for fiber enrichment and natural coloration, while carotenoid-rich carrot pomace, also characterized by the highest protein and ash contents, may contribute to nutritionally enhanced formulations. Plum pomace exhibited the highest content of individual phenolic compounds and the strongest antioxidant capacity, supporting its potential as a phenolic-rich functional ingredient. Apple pomace, characterized by chlorogenic acid, flavan-3-ols, and distinctive dihydrochalcones, may provide a complementary source of bioactive phenolics. Further studies should validate these applications in food matrices, focusing on techno-functional and sensory properties, phytochemical stability, and gastrointestinal bioaccessibility.

## Figures and Tables

**Table 1:** Proximate composition and color parameters.

Proximate Composition	BP	CP	PP	AP
Moisture (% dw)	12.22 ± 0.05 ^a^	11.21 ± 0.08 ^c^	11.97 ± 0.12 ^b^	10.50 ± 0.03 ^d^
Protein (% dw)	7.75 ± 0.21 ^b^	9.12 ± 0.05 ^a^	3.05 ± 0.03 ^d^	4.01 ± 0.02 ^c^
Ash (% dw)	5.45 ± 0.03 ^b^	7.78 ± 0.41 ^a^	2.78 ± 0.34 ^c^	1.84 ± 0.43 ^c^
Lipids (% dw)	0.12 ± 0.03 ^d^	1.28 ± 0.06 ^a^	0.59 ± 0.02 ^c^	1.04 ± 0.07 ^b^
Total fiber (% dw)	17.20 ± 0.55 ^a^	8.70 ± 0.31 ^b^	6.50 ± 0.19 ^c^	5.30 ± 0.64 ^c^
Total carbohydrates (% dw)	57.35 ± 0.85 ^d^	61.86 ± 0.72 ^c^	75.11 ± 0.91 ^d^	77.71 ± 0.65 ^a^
Color	BP	CP	PP	AP
L*	2.58 ± 0.04 ^c^	49.02 ± 0.05 ^a^	2.54 ± 0.08 ^c^	29.28 ± 0.09 ^b^
a*	88.85± 0.07 ^a^	12.08 ± 0.56 ^d^	60.38 ± 0.23 ^b^	21.90 ± 0.31 ^c^
b*	65.87 ± 0.21 ^c^	35.86 ± 0.13 ^d^	73.87 ± 0.11 ^b^	117.32 ± 0.71 ^a^

Data are expressed as the mean ± standard deviation (*n* = 3). Means within the same row followed by different superscript letters are significantly different (*p* < 0.05) according to Tukey’s honestly significant difference (HSD) test. BP, beetroot pomace; CP, carrot pomace; PP, plum pomace; AP, apple pomace; L*, lightness; a*, redness/greenness; b*, yellowness/blueness. Total carbohydrates calculated by difference.

**Table 2:** Phenolic and carotenoid profiles of extracted pomaces (μg/g dw).

Compound	Rt (min)	UV λmax (nm)	[M + H] + (*m*/*z*)	Subclass	CP	AP	PP	BP
2,3-Dihydroxybenzoic acid	2.73	270	155	Hydroxybenzoic acids	157.08 ± 0.32 ^b^	51.50 ± 0.51 ^c^	191.73 ± 0.23 ^a^	ND
3-Hydroxybenzoic acid	3.49	275	139		138.49 ± 0.21 ^a^	1.68 ± 0.24 ^d^	77.97 ± 0.44 ^b^	69.79 ± 0.12 ^c^
Gallic acid	5.01	280	171		52.24 ± 0.52 ^a^	ND	43.77 ± 0.27 ^b^	ND
Protocatechuic acid	9.15	290	155		29.61 ± 0.22 ^b^	ND	ND	142.80 ± 0.32 ^a^
3-Caffeoylquinic acid (Neochlorogenic)	9.98	330	355		17.87 ± 0.31 ^b^	ND	712.36 ± 0.31 ^a^	ND
5-Caffeoylquinic acid (Chlorogenic)	11.61	330	355		217.78 ± 0.34 ^b^	463.16 ± 0.35 ^a^	200.74 ± 0.52 ^c^	66.36 ± 0.21 ^d^
3-Feruloylquinic acid	8.63	322	369		148.90 ± 0.37 ^a^	ND	ND	ND
5-*p*-Coumaroylquinic acid	14.10	320	339		50.64 ± 0.11 ^a^	ND	ND	ND
3,5-Dicaffeoylquinic acid	17.54	330	517		16.54 ± 0.23 ^a^	ND	ND	ND
Caffeic acid	13.09	330	181		29.38 ± 0.23 ^b^	ND	ND	46.99 ± 0.21 ^a^
*p*-Coumaric acid	16.10	310	165		37.13 ± 0.13 ^a^	ND	ND	11.67 ± 0.43 ^b^
Quercetin-rutinoside (Rutin)	15.51	360, 255	611	Flavonols & flavanols	ND	149.02 ± 0.52 ^b^	309.78 ± 0.31 ^a^	ND
Quercetin-glucoside	16.37	360, 255	465		ND	64.00 ± 0.23 ^b^	232.74 ± 0.20 ^a^	ND
Quercetin-arabinoside	16.89	360, 256	435		ND	352.29 ± 0.18 ^a^	ND	ND
Quercetin-rhamnoside	17.40	360, 256	449		ND	79.23 ± 0.23 ^a^	ND	ND
Catechin	12.57	280	291		ND	105.83 ± 0.11 ^a^	ND	ND
Epicatechin	13.27	280	291		ND	129.15 ± 0.46 ^a^	ND	ND
Procyanidin dimer B1	13.66	280	579		ND	95.65 ± 0.27 ^a^	ND	ND
Procyanidin dimer B2	11.05	280	579		ND	81.86 ± 0.55 ^a^	ND	ND
Procyanidin trimer C1	14.09	280	867		ND	88.10 ± 0.32 ^a^	ND	ND
Cyanidin-rutinoside/glucoside	11.16	524, 280	595 449	Anthocyanins & Betacyanins	ND	ND	161.70 ± 0.28 ^a^	ND
Cyanidin-(caffeoyl-glucoside)	11.98	526, 330, 280	611		ND	ND	102.46 ± 0.22 ^a^	ND
Betanin-glucoside	2.65	535	713		ND	ND	ND	82.29 ± 0.23 ^a^
Betanin	7.83	536	389		ND	ND	ND	27.89 ± 0.53 ^a^
Betanidin	10.63	470	389		ND	ND	ND	103.89 ± 0.11 ^a^
Neobetanin	12.47	465	549		ND	ND	ND	67.50 ± 0.15 ^a^
Phloretin-xylosyl-glucoside	17.92	285	569	Dihydrochalcones	ND	84.81 ± 0.12 ^a^	ND	ND
Phloretin-glucoside (Phloridzin)	18.45	287	437		ND	13.28 ± 0.25 ^a^	ND	ND
Total phenolics					895.67 ± 0.25 ^c^	1759.57 ± 0.37 ^b^	5033.24 ± 0.28 ^a^	618.19 ± 0.18 ^d^
Compound								
Lutein	6.05	425, 446, 476		Carotenoids	22.78 ± 0.05 ^a^	1.14 ± 0.12 ^c^	11.57± 0.74 ^b^	0.000
β-Carotene	13.20	450, 480			61.59 ± 0.07 ^a^	5.24 ± 0.31 ^c^	21.26 ± 0.35 ^b^	3.28 ± 0.23 ^d^
**Carotenoids**					84.37 ± 0.08 ^a^	6.39 ± 0.32 ^c^	32.83 ± 0.81 ^b^	3.28 ± 0.23 ^d^

BP, beetroot pomace; CP, carrot pomace; PP, plum pomace; AP, apple pomace; ND, not detected; Values are expressed as mean ± standard deviation. Different superscript letters in the same column indicate statistically significant differences (*p* < 0.05) according to Tukey’s HSD test.

## Data Availability

The original contributions presented in the study are included in the article, further inquiries can be directed to the corresponding author.

## References

[1] FAO (Food and Agriculture Organization of the United Nations) (2026). FAOSTAT Statistical Database: Crops and Livestock Products. https://www.fao.org/faostat/en/#home.

[2] Milošević N., Glišić I., Đorđević M., Marić S., Radičević S., Milinković M., Tomić J., Mladenović J., Milošević T. (2026). Fruit Quality, Phenolic and Mineral Profiles of New Late-Season European Plum (*Prunus domestica* L.) Cultivars from Serbia. J. Food Compos. Anal..

[3] (2026). Eurostat Crop Production in EU Standard Humidity [Apro_cpsh1]. https://ec.europa.eu/eurostat/databrowser/view/apro_cpsh1__custom_22463521/default/table.

[4] Battistella Lasta H.F., Lentz L., Gonçalves Rodrigues L.G., Mezzomo N., Vitali L., Salvador Ferreira S.R. (2019). Pressurized Liquid Extraction Applied for the Recovery of Phenolic Compounds from Beetroot Waste. Biocatal. Agric. Biotechnol..

[5] Shoji T., Masumoto S., Miura T., Desjardins Y. (2024). Exploring the Potential of Apple (Poly)Phenols: A Systematic Review of Randomized Controlled Trials on Metabolic Diseases Prevention. Trends Food Sci. Technol..

[6] Ikram A., Rasheed A., Ahmad Khan A., Khan R., Ahmad M., Bashir R., Hassan Mohamed M. (2024). Exploring the Health Benefits and Utility of Carrots and Carrot Pomace: A Systematic Review. Int. J. Food Prop..

[7] Hong Y., Wang Z., Barrow C.J., Dunshea F.R., Suleria H.A.R. (2021). High-Throughput Screening and Characterization of Phenolic Compounds in Stone Fruits Waste by Lc-Esi-Qtof-Ms/Ms and Their Potential Antioxidant Activities. Antioxidants.

[8] Ahmed M., Das A.K., Roy O., Das S., Das K., Taher A., Aktaruzzaman M., Sarkar S., Koly J.S., Dipa R.A. (2026). Beetroot (*Beta vulgaris* L.) as a Functional Feed Additive in Poultry Nutrition: Implications for Sustainable Poultry Production. Poult. Sci..

[9] De La Fuente-Carmelino L., Anticona M., Ramos-Escudero F., Casimiro-Gonzales S., Muñoz A.M. (2025). Commercial Plant-Based Functional Beverages: A Comparative Study of Nutritional Composition and Bioactive Compounds. Beverages.

[10] Costa J.M., Ampese L.C., Ziero H.D.D., Sganzerla W.G., Forster-Carneiro T. (2022). Apple Pomace Biorefinery: Integrated Approaches for the Production of Bioenergy, Biochemicals, and Value-Added Products—An Updated Review. J. Environ. Chem. Eng..

[11] Rutyna A., Galus S. (2025). Valorisation of Carrot Pomace for Novel Crispbread Development Using the Potential of Chia Seeds (*Salvia hispanica* L.) as a Texturising Agent. Food Chem..

[12] Stoica F., Râpeanu G., Rațu R.N., Stănciuc N., Croitoru C., Țopa D., Jităreanu G. (2025). Red Beetroot and Its By-Products: A Comprehensive Review of Phytochemicals, Extraction Methods, Health Benefits, and Applications. Agriculture.

[13] Soares Mateus A.R., Pena A., Sendón R., Almeida C., Nieto G.A., Khwaldia K., Sanches Silva A. (2023). By-Products of Dates, Cherries, Plums and Artichokes: A Source of Valuable Bioactive Compounds. Trends Food Sci. Technol..

[14] Sheikh M.A., Saini C.S., Sharma H.K. (2023). Harnessing Plum (*Prunus domestica* L.) Processing Wastes for the Fabrication of Bio-Composite Edible Films: An Attempt towards a Food Circular Bioeconomy. Food Hydrocoll..

[15] Savic Gajic I.M., Savic I.M. (2023). Optimization of Ultrasound Procedure for the Sustainable Production of Oil from Plum Seeds. Carbon Resour. Convers..

[16] Katnić Đ., Porobić S.J., Lazarević-Pašti T., Kojić M., Tasić T., Marinović-Cincović M., Živojinović D. (2023). Sterilized Plum Pomace Biochar as a Low-Cost Effective Sorbent of Environmental Contaminants. J. Water Process Eng..

[17] Błaszczyk A., Sady S., Pachołek B., Jakubowska D., Grzybowska-Brzezińska M., Krzywonos M., Popek S. (2024). Sustainable Management Strategies for Fruit Processing Byproducts for Biorefineries: A Review. Sustainability.

[18] Lakshmikanthan M., Muthu S., Krishnan K., Altemimi A.B., Haider N.N., Govindan L., Selvakumari J., Alkanan Z.T., Cacciola F., Francis Y.M. (2024). A Comprehensive Review on Anthocyanin-Rich Foods: Insights into Extraction, Medicinal Potential, and Sustainable Applications. J. Agric. Food Res..

[19] Hafeez H.T., Muzammil H.S., Ahmad Z., Alsulami T., Waseem M., Mehmood T., Aadil R.M., Manzoor M.F., Abdi G. (2025). Assessment of Nutritional, Antioxidant, Physicochemical, and Storage Stability of Carrot Powder Supplemented Goat Milk Yogurt. J. Agric. Food Res..

[20] Wang H., Chen M., Li Y., Cui W., An Q., Yin X., Wang B. (2025). Exploring the Therapeutic Potential of Beetroot Juice in Patients with Peripheral Artery Disease: A Narrative Review. Nitric Oxide.

[21] Kaur A., Ghoshal G. (2025). Comprehensive Analysis of Phytochemical Extraction of Betalains from *Beta vulgaris* L. Pomace Using Conventional, Enzyme-Assisted and Ultrasonic-Assisted Methods. J. Food Meas. Charact..

[22] Arend G.D., Almeida É.S., Byruchko R.T., Pinto M.E.G., da Cruz A.B., Verruck S., Di Luccio M., Rezzadori K. (2022). Gravitational and Microwave-Assisted Multi-Stages Block Freeze Concentration Process to Obtain Enriched Concentrated Beet (*Beta vulgaris* L.) by-Products Extract: Bioactive Compounds and Simulated Gastrointestinal Profile. Food Bioprod. Process..

[23] Nirmal N.P., Khanashyam A.C., Mundanat A.S., Shah K., Babu K.S., Thorakkattu P., Al-Asmari F., Pandiselvam R. (2023). Valorization of Fruit Waste for Bioactive Compounds and Their Applications in the Food Industry. Foods.

[24] Antonić B., Jančíková S., Dordević D., Tremlová B. (2020). Grape Pomace Valorization: A Systematic Review and Meta-Analysis. Foods.

[25] Curutchet A., Trias J., Tárrega A., Arcia P. (2021). Consumer Response to Cake with Apple Pomace as a Sustainable Source of Fibre. Foods.

[26] Vandorou M., Plakidis C., Tsompanidou I.M., Adamantidi T., Panagopoulou E.A., Tsoupras A. (2024). A Review on Apple Pomace Bioactives for Natural Functional Food and Cosmetic Products with Therapeutic Health-Promoting Properties. Int. J. Mol. Sci..

[27] Mistrianu S.L., Constantin O.E., Horincar G., Andronoiu D.G., Stănciuc N., Muresan C., Râpeanu G. (2022). Beetroot By-Product as a Functional Ingredient for Obtaining Value-Added Mayonnaise. Processes.

[28] Rațu R.N., Oncică G.F., Stoica F., Constantin O.E., Stănciuc N., Aprodu I., Andronoiu D.G., Banožić M., Ćujić Nikolić N., Râpeanu G. (2025). Value-Added Carp Roe Salad Supplemented with Orange Carrot Pomace Powder. Foods.

[29] Kumar S., Yadav S., Rani R., Pathera A.K. (2024). Effects of Plum Powder and Apple Pomace Powder Addition on the Physico-Chemical, Sensory, and Textural Properties of Buffalo Meat Emulsion. Nutr. Food Sci..

[30] Kultys E., Moczkowska-Wyrwisz M. (2022). Effect of Using Carrot Pomace and Beetroot-Apple Pomace on Physicochemical and Sensory Properties of Pasta. LWT.

[31] Lima de Souza R., Pérez-Gago M.B., Palou L. (2026). From Waste to Health: Valorization of Agri-Food by-Products for the Control of Fresh Fruit Fungal Postharvest Decay. Postharvest Biol. Technol..

[32] Gil-Martín E., Forbes-Hernández T., Romero A., Cianciosi D., Giampieri F., Battino M. (2022). Influence of the Extraction Method on the Recovery of Bioactive Phenolic Compounds from Food Industry By-Products. Food Chem..

[33] Fărcaș A.C., Socaci S.A., Chiș M.S., Dulf F.V., Podea P., Tofană M. (2022). Analysis of Fatty Acids, Amino Acids and Volatile Profile of Apple By-Products by Gas Chromatography-Mass Spectrometry. Molecules.

[34] (2000). American Association of Cereal Chemists Method 38-12, 44-15.02, 46-11.02, 30-25.01, 08-01.01, 32-07.01. Approved Methods of the American Association of Cereal Chemists.

[35] Man S.M., Pǎucean A., Cǎlian I.D., Murean V., Chi M.S., Pop A., Murean A.E., Bota M., Muste S. (2019). Influence of Fenugreek Flour (*Trigonella foenum-graecum* L.) Addition on the Technofunctional Properties of Dark Wheat Flour. J. Food Qual..

[36] Igual M., Chiş M.S., Păucean A., Vodnar D.C., Ranga F., Mihăiescu T., Török A.I., Fărcaș A., Martínez-Monzó J., García-Segovia P. (2021). Effect on Nutritional and Functional Characteristics by Encapsulating Rose Canina Powder in Enriched Corn Extrudates. Foods.

[37] Chiş M.S., Păucean A., Man S.M., Vodnar D.C., Teleky B.-E., Pop C.R., Stan L., Borsai O., Kadar C.B., Urcan A.C. (2020). Quinoa Sourdough Fermented with Lactobacillus Plantarum ATCC 8014 Designed for Gluten-Free Muffins—A Powerful Tool to Enhance Bioactive Compounds. Appl. Sci..

[38] Neveu V., Perez-Jimenez J., Vos F., Crespy V., du Chaffaut L., Mennen L., Knox C., Eisner R., Cruz J., Wishart D. (2010). Phenol-Explorer: An Online Comprehensive Database on Polyphenol Contents in Foods. Database.

[39] Szabo K., Dulf F.V., Teleky B.-E., Eleni P., Boukouvalas C., Krokida M., Kapsalis N., Rusu A.V., Socol C.T., Vodnar D.C. (2021). Evaluation of the Bioactive Compounds Found in Tomato Seed Oil and Tomato Peels Influenced by Industrial Heat Treatments. Foods.

[40] Agudelo C., Igual M., Camacho M., Martínez-Navarrete N. (2017). Effect of Process Technology on the Nutritional, Functional, and Physical Quality of Grapefruit Powder. Food Sci. Technol. Int..

[41] Crişan D., Frumuzachi O., Babotă M., Gavrilaş L., Ranga F., Mocan A., Vodnar D.C., Crişan G. (2025). Phytochemical Variability and Bioactive Potential of Sambucus Nigra L. Flower Extracts: A Comparative Study across Four Ecological Regions from Romania. Ind. Crops Prod..

[42] Ferreira D.M., Machado S., Espírito Santo L., Costa A.S.G., Ranga F., Chiș M.S., Palmeira J.D., Oliveira M.B.P.P., Alves R.C., Ferreira H. (2024). Changes in the Composition of Olive Pomace after Fermentation: A Preliminary Study. Fermentation.

[43] Tay J.B.J., Chua X., Ang C., Subramanian G.S., Tan S.Y., Lin E.M.J., Wu W.Y., Goh K.K.T., Lim K. (2021). Effects of Spray-Drying Inlet Temperature on the Production of High-Quality Native Rice Starch. Processes.

[44] Antonic B., Jancikova S., Dordevic D., Tremlova B. (2020). Apple Pomace as Food Fortification Ingredient: A Systematic Review and Meta-Analysis. J. Food Sci..

[45] Sahni P., Shere D.M. (2017). Comparative Evaluation of Physico-Chemical and Functional Properties of Apple, Carrot and Beetroot Pomace Powders. Intl. J. Food. Ferment. Technol..

[46] Ceclu L., Corbu A.R., Rumeus I., Cicanci A., Blejan A.M., Vîșcu I., Nour V. (2026). Thin-Layer Drying Kinetics and Quality Attributes of Apple Pomace Powders from Different Varieties. Foods.

[47] Milala J., Kosmala M., Sójka M., Kołodziejczyk K., Zbrzeåniak M., Markowski J. (2013). Plum Pomaces as a Potential Source of Dietary Fibre: Composition and Antioxidant Properties. J. Food Sci. Technol..

[48] Luca M.I., Ungureanu-Iuga M., Mironeasa S. (2022). Carrot Pomace Characterization for Application in Cereal-Based Products. Appl. Sci..

[49] Costa A.P.D., Hermes V.S., Rios A.O., Flôres S.H. (2017). Minimally Processed Beetroot Waste as an Alternative Source to Obtain Functional Ingredients. J. Food Sci. Technol..

[50] Swastike W., Suryanto E., Rusman R., Hanim C., Jamhari J., Erwanto Y., Jumari J. (2020). The Substitution Effects of Tapioca Starch and Beetroot Powder as Filler on the Physical and Sensory Characteristics of Chicken Sausage. J. Ilmu Dan Teknol. Has. Ternak.

[51] Gracey P.R., Tako E. (2025). Apple and Grape Pomace: Emerging Upcycled Functional Ingredients in Processed Meat Products, Designed to Increase Polyphenol and Fiber Contents. Sustain. Food Technol..

[52] Ahmed M., Zhang H., Xiong Y. (2026). Anthocyanin Structural Types and Stability: Implications for Natural Colorant Applications in Food Systems. Foods.

[53] Eliopoulos C., Markou G., Langousi I., Arapoglou D. (2022). Reintegration of Food Industry By-Products: Potential Applications. Foods.

[54] Kandemir K., Piskin E., Xiao J., Tomas M., Capanoglu E. (2022). Fruit Juice Industry Wastes as a Source of Bioactives. J. Agric. Food Chem..

[55] Domínguez R., Munekata P.E.S., Pateiro M., Maggiolino A., Bohrer B., Lorenzo J.M. (2020). Red Beetroot. A Potential Source of Natural Additives for the Meat Industry. Appl. Sci..

[56] Bashmil Y.M., Dunshea F.R., Appels R., Suleria H.A.R. (2024). Bioaccessibility of Phenolic Compounds, Resistant Starch, and Dietary Fibers from Australian Green Banana during In Vitro Digestion and Colonic Fermentation. Molecules.

[57] Spence C. (2015). On the Psychological Impact of Food Colour. Flavour.

[58] Fu Y., Shi J., Xie S.Y., Zhang T.Y., Soladoye O.P., Aluko R.E. (2020). Red Beetroot Betalains: Perspectives on Extraction, Processing, and Potential Health Benefits. J. Agric. Food Chem..

[59] Benucci I., Lombardelli C., Mazzocchi C., Esti M. (2022). Natural Colorants from Vegetable Food Waste: Recovery, Regulatory Aspects, and Stability—A Review. Compr. Rev. Food Sci. Food Saf..

[60] Gorjanović S., Micić D., Pastor F., Tosti T., Kalušević A., Ristić S., Zlatanovic S. (2020). Evaluation of Apple Pomace Flour Obtained Industrially by Dehydration as a Source of Biomolecules with Antioxidant, Antidiabetic and Antiobesity Effects. Antioxidants.

[61] Tumbas Šaponjac V., Čanadanović-Brunet J., Ćetković G., Jakišić M., Djilas S., Vulić J., Stajčić S. (2016). Encapsulation of Beetroot Pomace Extract: RSM Optimization, Storage and Gastrointestinal Stability. Molecules.

[62] Bas-Bellver C., Barrera C., Betoret N., Seguí L. (2023). Effect of Processing and In Vitro Digestion on Bioactive Constituents of Powdered IV Range Carrot (*Daucus carota*, L.) Wastes. Foods.

[63] Gurev A., Cesko T., Dragancea V., Ghendov-Mosanu A., Pintea A., Sturza R. (2023). Ultrasound- and Microwave-Assisted Extraction of Pectin from Apple Pomace and Its Effect on the Quality of Fruit Bars. Foods.

[64] Gorjanović S., Pastor F.T., Micić D., Dodevska M., Ristić S., Petričević S., Dujmić F., Zlatanović S. (2026). Valorization of Beetroot Pomace as a Flour Fortifier, Functional Ingredient and Dietary Supplement. Foods.

[65] Rabeeah I., Gruber-Schmidt V., Murray H., Afsharzadeh N., Paltram R., Marinovic S., Zia H., Hutabarat O.S., Hofsommer M., Slatnar A. (2024). Apple Pomace as a Potential Source of Oxidative Stress-Protecting Dihydrochalcones. Antioxidants.

[66] Mahdi M., Jasim A. (2025). Black Plum Peels Aqueous Extract: A Natural Source of Antioxidants and Antibacterial Agents. Turk. J. Agric.-Food Sci. Technol..

[67] Ciccoritti R., Ciorba R., Ceccarelli D., Amoriello M., Amoriello T. (2024). Phytochemical and Functional Properties of Fruit and Vegetable Processing By-Products. Appl. Sci..

[68] Zeng Y., Zhou W., Yu J., Zhao L., Wang K., Hu Z., Liu X. (2023). By-Products of Fruit and Vegetables: Antioxidant Properties of Extractable and Non-Extractable Phenolic Compounds. Antioxidants.

[69] Tiliwa E.S., Boateng I.D., Zhou C., Xu B. (2023). Role of Drying Technologies on the Drying Kinetics, Physical Quality, Aroma, and Enzymatic Activity of Pineapple Slices. Sustainability.

[70] Arruda H.S., Silva E.K., Araujo N.M.P., Pereira G.A., Pastore G.M., Junior M.R.M. (2021). Anthocyanins Recovered from Agri-Food by-Products Using Innovative Processes: Trends, Challenges, and Perspectives for Their Application in Food Systems. Molecules.

[71] Moulick S.P., Jahan F., Islam M.B., Al Bashera M., Hasan M.S., Islam M.J., Ahmed S., Karmakar D., Ahmed F., Saha T. (2023). Nutritional Characteristics and Antiradical Activity of Turmeric (*Curcuma longa* L.), Beetroot (*Beta vulgaris* L.), and Carrot (*Daucus carota* L.) Grown in Bangladesh. Heliyon.

[72] Michalska A., Wojdyło A., Majerska J., Lech K., Brzezowska J. (2019). Qualitative and Quantitative Evaluation of Heat-Induced Changes in Polyphenols and Antioxidant Capacity in *Prunus domestica* L. By-Products. Molecules.

[73] Luca M.I., Ungureanu-Iuga M., Mironeasa S. (2024). Nutritional and Bioactivecomposition of Different Carrot Pomace Varieties. Ukr. J. Food Sci..

[74] Ziobro R., Gumul D., Sabat R., Wywrocka-Gurgul A., Zięba T. (2026). Effect of Addition Levels of By-Product Mixture (Apple Pomace: Red Potato Pulp: Sugar Beet Pulp) on Phytochemical Profile, Antioxidant Activity and Physical Properties of Extruded Corn Snacks. Molecules.

[75] Enaru B., Drețcanu G., Pop T.D., Stǎnilǎ A., Diaconeasa Z. (2021). Anthocyanins: Factors Affecting Their Stability and Degradation. Antioxidants.

[76] Shahidi F., Hossain A. (2023). Importance of Insoluble-Bound Phenolics to the Antioxidant Potential Is Dictated by Source Material. Antioxidants.

[77] Arnao M.B. (2000). Some Methodological Problems in the Determination of Antioxidant Activity Using Chromogen Radicals: A Practical Case. Trends Food Sci. Technol..

[78] Ziobro R., Ivanišová E., Bojňanská T., Gumul D. (2022). Retention of Antioxidants from Dried Carrot Pomace in Wheat Bread. Appl. Sci..

[79] Platzer M., Kiese S., Herfellner T., Schweiggert-Weisz U., Miesbauer O., Eisner P. (2021). Common Trends and Differences in Antioxidant Activity Analysis of Phenolic Substances Using Single Electron Transfer Based Assays. Molecules.

[80] Francenia Santos-Sánchez N., Salas-Coronado R., Villanueva-Cañongo C., Hernández-Carlos B. (2019). Antioxidant Compounds and Their Antioxidant Mechanism. Antioxidants.

[81] Yener E., Saroglu O., Sagdic O., Karadag A. (2023). The Effects of Different Drying Methods on the In Vitro Bioaccessibility of Phenolics, Antioxidant Capacity, and Morphology of European Plums (*Prunes domestica* L.). ACS Omega.

[82] Gumul D., Ziobro R., Korus J., Kruczek M. (2021). Apple Pomace as a Source of Bioactive Polyphenol Compounds in Gluten-Free Breads. Antioxidants.

[83] Hobbi P., Okoro O.V., Hajiabbas M., Hamidi M., Nie L., Megalizzi V., Musonge P., Dodi G., Shavandi A. (2023). Chemical Composition, Antioxidant Activity and Cytocompatibility of Polyphenolic Compounds Extracted from Food Industry Apple Waste: Potential in Biomedical Application. Molecules.

[84] Oras A., Akagić A., Spaho N., Gaši F., Žuljević S.O., Meland M. (2023). Distribution and Stability of Polyphenols in Juices Made from Traditional Apple Cultivars Grown in Bosnia and Herzegovina. Molecules.

[85] Ciurlă L., Enache I.M., Buțerchi I., Mihalache G., Lipșa F.D., Patraș A. (2025). A New Approach to Recover Bioactive Compounds from Apple Pomace: Healthy Jelly Candies. Foods.

[86] Bindon K., Qi S., Kassara S., Nicolotti L., Jouin A., Beer M. (2023). Apple Pomace Compositional Data Highlighting the Proportional Contribution of Polymeric Procyanidins. Molecules.

[87] Szabo K., Mitrea L., Călinoiu L.F., Teleky B.E., Martău G.A., Plamada D., Pascuta M.S., Nemeş S.A., Varvara R.A., Vodnar D.C. (2022). Natural Polyphenol Recovery from Apple-, Cereal-, and Tomato-Processing By-Products and Related Health-Promoting Properties. Molecules.

[88] Desseva I., Stoyanova M., Petkova N., Mihaylova D. (2020). Red Beetroot Juice Phytochemicals Bioaccessibility: An in Vitro Approach. Pol. J. Food Nutr. Sci..

[89] Trych U., Buniowska-Olejnik M., Marszałek K. (2022). Bioaccessibility of Betalains in Beetroot (*Beta vulgaris* L.) Juice under Different High-Pressure Techniques. Molecules.

[90] Wang Z., Li S., Ge S., Lin S. (2020). Review of Distribution, Extraction Methods, and Health Benefits of Bound Phenolics in Food Plants. J. Agric. Food Chem..

[91] Ospina-Posada A.C., Porras O., Rincón-Cervera M.A., Frias J., Zielinski A.A.F., Bridi R., Arias-Santé M.F., de Camargo A.C. (2024). Antioxidant Properties of Phenolic Extracts of Murtilla Pomace: First Report on the Importance of Soluble and Insoluble-Bound Compounds. Food Res. Int..

[92] Pérez-gálvez A., Viera I., Roca M. (2020). Carotenoids and Chlorophylls as Antioxidants. Antioxidants.

[93] Richards J., Lammert A., Madden J., Kang I., Amin S. (2024). Physical Treatments Modified the Functionality of Carrot Pomace. Foods.

[94] Molteni C., La Motta C., Valoppi F. (2022). Improving the Bioaccessibility and Bioavailability of Carotenoids by Means of Nanostructured Delivery Systems: A Comprehensive Review. Antioxidants.

[95] Vaz A.A., Odriozola-Serrano I., Oms-Oliu G., Martín-Belloso O. (2022). Physicochemical Properties and Bioaccessibility of Phenolic Compounds of Dietary Fibre Concentrates from Vegetable By-Products. Foods.

[96] Hidalgo A., Brandolini A., Čanadanović-Brunet J., Ćetković G., Tumbas Šaponjac V. (2018). Microencapsulates and Extracts from Red Beetroot Pomace Modify Antioxidant Capacity, Heat Damage and Colour of Pseudocereals-Enriched Einkorn Water Biscuits. Food Chem..

[97] Gołębiewska E., Kalinowska M., Yildiz G. (2022). Sustainable Use of Apple Pomace (AP) in Different Industrial Sectors. Materials.

[98] Yao J., Chen W., Fan K. (2023). Novel Efficient Physical Technologies for Enhancing Freeze Drying of Fruits and Vegetables: A Review. Foods.

[99] Chang L.S., Karim R., Abdulkarim S.M., Yusof Y.A., Ghazali H.M. (2018). Storage Stability, Color Kinetics and Morphology of Spray-Dried Soursop (*Annona muricata* L.) Powder: Effect of Anticaking Agents. Int. J. Food Prop..

